# Epidemiology of Campus Football Injuries in Ningxia, China: Occurrence, Causes, and Management

**DOI:** 10.3389/fpubh.2022.893541

**Published:** 2022-04-26

**Authors:** Hengyuan Liu, Sen Huang, Te Bu, Wei Jiang, Tao Fu, Liliang Zhao

**Affiliations:** ^1^College of Physical Education, Hunan Normal University, Changsha, China; ^2^Hunan Institute of Sport Science, Changsha, China; ^3^College of Education, Ningxia University, Yinchuan, China; ^4^College of Exercise and Health Sciences, Tianjin University of Sport, Tianjin, China

**Keywords:** soccer, youth athletes, injury prevention, first aid, risk factors, healthcare

## Abstract

**Objective:**

By 2022, the Chinese government intends to have more than 30 million primary-, middle- and high-school children and adolescents regularly participate in campus football. In contrast, epidemiology of campus football injuries is completely missing. The goal of this descriptive epidemiological study was to determine the current state of campus football injuries and then to recommend appropriate prevention and management strategies.

**Methods:**

This retrospective epidemiological study conducted a survey of students, physical education and football teachers in primary, middle and high schools in the Ningxia Autonomous Region to determine the campus football injuries that occurred throughout the preceding 12-month period. The survey comprised questions on demographic characteristics, the occurrence, causes, and management of campus football injuries.

**Results:**

A total of 1,285 students and 200 teachers returned eligible surveys. 25.7% of students had encountered injury accidents while participating in campus football activities. 31.3% of high school students, 23.8% of middle school students, and 19.2% of primary school students have sustained injuries. Football competition, accounting for 45.4% of all injuries, is the leading cause of injury. Football class teaching, which accounted for 3.0% of all injuries, had the lowest injury rate of any campus football activity. Students and teachers reported that a lack of safety awareness and injury prevention education were the primary causes of injuries. Only 18.7% and 11.4% of students are familiar with first aid basics and cardiopulmonary resuscitation, respectively. 10.6% and 7.5% of students lack any first aid basics and skills, respectively. 43.9% of students lack insurance coverage for athletic injuries. 62.5% and 38.5% of teachers reported that schools lack first aid training and an emergency plan for injuries, respectively.

**Conclusion:**

Students in Ningxia's campus football programs have a high injury risk. Injury prevention and management strategies lag significantly behind the mainstream nationwide promotion of campus football in China.

## Introduction

Football (soccer) is the most popular sport in the world, with an estimated 4 billion fans worldwide (1). Despite concerns about the safety of youth football training, a growing body of evidence indicates that regular participation in well-designed, rationally advanced, and professionally instructed football training programs can provide children and adolescents with substantial health and fitness advantages (2, 3). On March 16, 2015, the State Council announced the “General plan of Chinese football reform and development” (4), ushering in a new age of “Campus Football 2.0” in China. Since the Chinese government implemented a series of comprehensive plans and guidelines, campus football has grown rapidly in China. In Chongqing for example, the number of students who participate in campus football activities on a regular basis has nearly doubled from <20,000 in 2014 to more than 400,000 in 2016, a nearly 20-fold increase (5). On August 28, 2020, the Ministry of Education published a “Notice of Action Plan for the Construction of the Eight Major Systems of National Youth Campus Football” (6). The Action Plan establishes a working target of more than 30 million primary-, middle-, and high-school students participating in football on a regular basis by 2022. After establishing the framework for campus football, the directive objectives and improvement plans for the construction of the “Eight Major Systems” were presented. China has essentially finished the five-in-one national campus football promotion pattern of “distinctive schools + high-level football teams in colleges and universities + pilot counties (districts) + reform pilot zones + Man Tian Xing training camps.” By 2021, the “multiple pillar” of campus football reform had been largely completed, with 2,038 schools, 2,030 kindergartens, 40 pilot zones, five pilot counties (districts), and 28 “Man Tian Xing” training camps established for campus football nationwide (7). Campus football activities are gaining huge popularity throughout China.

Apart from the increased interest and engagement in campus football across the nation, the game itself is a confrontation sport that requires rapid acceleration and deceleration, direction changes, and jumping and landing challenges. Due to the frequent physical contact involved, the risk of injury is significant (8). Injury risk is especially significant in youth sports, where the growth and maturation of children and adolescents may predispose youth players to an increased risk, and football has been shown to have the highest occurrence and severity of injuries among popular youth sports (9). As campus football activities have grown in popularity, an increasing number of injuries have occurred (10). On the one hand, high incidence rates impose significant socioeconomic consequences on the public healthcare system (11, 12). On the other side, major injuries can have a long-term effect on an individual player's health (11). Children and adolescents who sustain sports injuries may experience significant reductions in their current and future participation in physical activities (11), which is directly contrary to the promotion of campus football in China. As a result, it is critical to assess epidemiological data during campus football participation. A systematic examination of occurrence and risk factors enables the development of effective preventative measures, ensuring the healthy development of youth and youth sports as a whole.

Athletic injuries have severely hampered the growth of campus football in China (13, 14). With the rapid growth of campus football, it is vital to analyze epidemiological data in order to design appropriate safety education programs tailored for Chinese youth and schools. Using a representative sample who participated in campus football programs in Ningxia, this study aimed to determine the occurrence, causes, and management of campus football injuries. The discussion and findings are likely to aid in the long-term development of campus football.

## Methods

### Participants

The Ningxia Autonomous Region in China was chosen as a sample region for campus football injuries in this study. The sample was drawn from a convenience sample who participated in the Ministry of Education certified campus football programs in the preceding year. This study included students, physical education (PE) teachers and football coaches (hereafter, referred as teachers) from primary, middle and high schools. The ethics committee of Ningxia University approved the study.

### Instrument

The data collection instrument was a self-administered, anonymous paper-and-pencil survey (“Research Questionnaire on Campus Football Safety Prevention System,” in Chinese). Two versions were developed to meet the research aims for students and teachers, respectively. An injury in the surveys was defined as one that required a teacher to enter the field to assess a player's condition, or one that required a player to withdraw from participation, or one that required any type of first aid during an incident that occurred throughout the preceding 12-month period. Existing football injuries survey modules from the literature (15–17) were used to construct the surveys.

Occurrence of campus football injuries was assessed by one item: “Have you ever been hurt while participating in campus football activities? A. Yes B. No” (students survey).

Causes of campus football injuries were assessed by five items: (1) “Which of the following activities resulted in your injuries while playing campus football? How many times has each activity resulted in an injury in the past academic year? (multiple choice) A. Football class teaching (on-campus, supervised sessions) B. Football competition (on-campus, supervised sessions) C. Extra-curricular activities (on-campus, unsupervised sessions) D. After-school football training (off-campus, supervised sessions) E. After-school football activities (off-campus, unsupervised sessions)” (students survey); (2) “What, in your opinion, are the safety factors affecting the development of campus football activities? (multiple choice) A. Lack of safety knowledge on the part of students B. Inadequate safety awareness and behavior among students C. Poor physical characteristics of students D. Lack of safety knowledge on the part of teachers E. Inadequate safety awareness of teachers F. Inadequate classroom management by teachers G. Teachers' lack of seriousness and accountability in class H. Teachers' exercise schedules are excessive J. Sports equipment and pitch present safety issues K. Inclement weather L. Inadequate safety management of the school” (students survey); (3) “What factors do you believe contribute to acute injuries among students? (multiple choice) A. Inadequate warm-up B. Inclement weather C. Poor physical characteristics of students D. Excessive risk-taking among students E. Errors in technical movement of students F. Sports equipment and pitch present safety issues G. Lack of self-protection skills and safety knowledge on the part of students H. Lack of professional knowledge on the part of teachers I. Inadequate safety awareness of teachers” (teachers survey); (4) “Have your teachers taught you the methods of self-protection in sports? A. Often taught B. Occasionally taught C. Rarely taught D. Did not teach” (students survey); and (5) “Is the pitch and equipment sufficient for your campus football activities? A. Fulfill the requirements B. Fulfill only the basic requirements C. Could not fulfill the requirements” (students survey).

Management of campus football injuries was assessed by six items: (1) “Do you know first aid basics in the event of an athletic injury accident? A. Clear understanding B. Partial understanding C. No understanding” (students survey); (2) “Is there a first aid course offered at your school? A.Yes B.No” (teachers survey); (3) “Do you know how to administer the following first aid skills (multiple choice): A. Ice compression B. Simple bandage C. Immobilization of the torso and limbs D. Cardiopulmonary resuscitation E. No knowledge of basic first aid” (students survey); (4) “If a financial disagreement emerges as a result of an athletic injury sustained while participating in campus football activities, who do you believe should be held accountable: A. The school B. Personal responsibility C. A third party, such as a healthcare insurance” (students survey); (5) “Do you have insurance for athletic injuries? A.Yes B.No” (students survey); and (6) “Is there an emergency plan in place at your school for athletic injuries sustained during campus football activities? A.Yes B.No” (teachers survey).

### Data Collection

In classrooms at the schools, research assistants contacted potential volunteers. To those who expressed an interest, a consent letter and survey instrument were distributed. Each participant provided written consent. In order to collect as accurate data as possible, research assistants explicitly explained all items in the survey to the participants. If participants had any queries concerning the items in the surveys, researcher assistants and/or students' parents clarified them before they responded. Students and teachers who agreed to participate completed a paper survey and returned it to a classroom research assistant.

### Statistics

Two researchers independently entered the data into Excel (Microsoft Corporation, USA), and if there were discrepancies, a third researcher verified the accuracy of the data. Descriptive statistics were performed (SPSS, IBM, USA) to describe the characteristics of survey items.

## Results

### Participants

A total of 1,362 surveys were sent out to students, all of which were returned and 1,285 were effective, with an effective rate of 94.3%. Table 1 shows the demographic data of students participating in campus football injuries survey. A total of 213 surveys were sent out to teachers, all of them were returned, and 200 were effective, with an effective rate of 93.9%.

**Table 1:** Demographic data of students of various grades.

**Grade**	**Male**		**Female**		**Missing sex data**		**Overall**	
	**Age (years)**	**N**	**Age (years)**	**N**	**Age (years)**	**N**	**Age (years)**	**N**
High school	16.0 ± 1.2	336	15.6 ± 1.2	187	15.9 ± 1.2	11	15.9 ± 1.2	534
Middle school	13.6 ± 1.0	239	13.6 ± 1.0	164	13.0 ± 0.0	4	13.6 ± 1.0	407
Primary school	11.3 ± 1.1	192	11.4 ± 0.8	146	12.0 ± 0.0	6	11.3 ± 1.0	344

*Data are expressed as means ± standard deviations*.

### Occurrence of Campus Football Injuries in Ningxia

According to the results of an effective survey distributed to 1,285 students in Ningxia (Table 2), 330 students sustained injuries while participating in campus football activities, accounting for 25.7% of the total respondents. Risks of injury tend to increase as a student progresses through primary school, middle school, and high school campus football activities.

**Table 2:** Injury occurrence during campus football activities.

**Whether or not injuries had occurred**	**High school (n**_1_ **)**		**Middle school (n**_2_ **)**		**Primary school (n**_3_ **)**		**Overall (n**_4_ **)**	
	**YES**	**NO**	**YES**	**NO**	**YES**	**NO**	**YES**	**NO**
N	167	367	97	310	66	278	330	955
%	31.3	68.7	23.8	76.2	19.2	80.8	25.7	74.3

*n_1_ = 534, n_2_ = 407, n_3_ = 344, n_4_ = 1,285*.

### Causes of Campus Football Injuries in Ningxia

Table 3 summarizes football activities that resulted in campus football injuries. Football competition is the primary activity among the five forms of football activities in which injuries occur to students of all school grades. Football class teaching had the lowest proportion of injuries.

**Table 3:** Football activities that result in campus football injuries.

**Forms of football activities**	**High school (n**_**1**_ **)**		**Middle school (n**_**2**_ **)**		**Primary school (n**_**3**_ **)**		**Overall (n**_**4**_ **)**	
	**N**	**%**	**N**	**%**	**N**	**%**	**N**	**%**
Football class teaching	16	3.0	16	4.0	6	1.8	38	3.0
Football competition	274	51.6	169	42.7	132	38.9	575	45.4
Extra-curricular football activities	98	18.5	64	16.2	76	22.4	238	18.8
After-school football training	38	7.2	37	9.3	49	14.5	124	9.8
After-school football activities	105	19.8	110	27.8	76	22.4	291	23.0

*n_1_ = 531, n_2_ = 396, n_3_ = 339, n_4_ = 1,266*.

According to a student survey, 80.9% believed that the cause of injury accidents was students' own lack of safety knowledge, 71.2% believed that students' lack of safety awareness and behavior contributed to the occurrence of injury accidents, and 54.8% believed that students' poor physical characteristics contributed to the occurrence of injury accidents.

According to a teacher survey, 61.0% believed that the cause of injury accidents was students' lack of self-protection skills and safety knowledge, 42.0% believed that inadequate safety awareness of teachers contributed to student injuries, and 33.0% believed that teachers' lack of professional knowledge contributed to student injuries. Additionally, 62.0% of teachers often taught students self-protection techniques during campus football activities, 30.5% occasionally taught students self-protection techniques, and 7.5% seldom or never taught students self-protection techniques.

According to the surveys on the causes of student injuries, 32.2% of students and 38.0% of teachers cited safety issues on sports equipment and pitch as the cause of injury accidents. According to a survey of students' satisfaction with the pitch and equipment used for campus football activities, 42.7% of students believed the school pitch and equipment could meet the needs of sports, 46.7% believed they could only meet the basic needs of sports, and 10.6% believed they could not meet the needs of sports.

### Management of Campus Football Injuries in Ningxia

First aid is applicable to a wide variety of medical circumstances and entails both specialized knowledge and skills as well as the ability to assess and make appropriate decisions. Only 18.7% of students are aware of first aid basics, while 10.6% are completely unaware. 70.7% of students have a hazy understanding of first aid basics. The results of a teacher survey indicate that 62.5% of schools lack first aid training.

According to the results of a student survey (Table 4), 52.1% of students knew how to apply ice compression in the event of an injury, 40.1% knew how to apply simple bandage techniques, and 12.1% knew how to immobilize the torso and limbs when treating severe injuries. Only 11.4% of students are familiar with cardiopulmonary resuscitation (CPR). 7.5% of students are unaware of any first aid skills.

**Table 4:** Students' proficiency of first aid skills.

**First aid proficiency**	**N**	**%**
Ice compression	669	52.1
Simple bandage	515	40.1
Immobilization	155	12.1
Cardiopulmonary resuscitation	147	11.4
Know nothing	96	7.5

According to the survey of solutions to campus football injuries, 16.4% of students believe that the school should bear responsibility, 23.3% believe that students should bear responsibility, and 60.2% believe that a third party, such as an insurance company, should bear responsibility. Additionally, 43.9% of students lack health insurance coverage for athletic injuries. Finally, 38.5% of teachers reported that schools lack an emergency plan for student injuries sustained during campus football activities.

## Discussion

This retrospective epidemiological study was conducted to identify football injury situations in Ningxia, China. To our knowledge, this is the first study in China to examine the epidemiology of campus football injuries in a systematic manner. Our findings indicate that there is a high occurrence of student injuries associated with both independent student participation in campus football activities and instructor participation in campus football activities during teaching sessions. Injuries sustained during campus football activities have a direct impact on the development of campus football in China as well as the health and growth of children and adolescents. Therefore, enhancing the prevention and management of campus football injuries should become a priority for organizing campus football.

First, our results indicate that campus football injuries occur at a rate of 25.7% among 1,285 students in Ningxia, which are relatively higher than those as a result of general PE classes. For example, Ding surveyed 1,300 students in 26 middle schools from Wuxi, China (18). It was discovered that athletic injuries occurred at a rate of 18.1% during PE classes. Likewise, Wu and Zhang (19) surveyed 1,570 students in middle schools in Zhejiang Province. Their findings indicated that students who had sustained athletic injuries during PE classes accounted for 16% of the total. In general, youth football exposes a greater risk of injury than many other sports (9, 20), because the game itself demands a higher level of playing intensity, a greater volume of training, stronger players, heightened competitiveness, and an aggressive playing style. As a result, we recommend designing campus football injury prevention education programs that incorporate practical injury prevention training to children and adolescents, in order to raise awareness about injury risk and minimize injury occurrence.

Second, our results indicate that the risk of injury tends to increase with increasing school grade levels. This is consistent with results from developed football nations indicating age-related disparities in injury patterns. A prospective descriptive epidemiological research of football injuries over two seasons in the Czech Republic and Switzerland found that injury occurrence rose with increasing age among youth players aged 7 to 12 years (21). A recent prospective cohort study conducted at a national youth football academy (U13–U18; age range: 11–18 years) also discovered that injury occurrence was greater among older players and more than doubled between the U13 and U18 age groups (22). Middle- and high-school students' athletic ability has increased with age, and they participate in more intensive physical activities than primary-school students (23, 24), which adds to the higher injury occurrence among middle- and high-school students. It should be noted that injury occurrence could have a lasting impact on the youth growth as well as the promotion of campus football. It was shown that having a history of injury in youth was associated with a higher likelihood of injury (recurrent injury) as children and adolescents grow (25, 26), which could result in youth's increased financial obligations (27) and an earlier end to football participation (11). In light of this characteristic, all segments of society should take appropriate steps to mitigate youth injury in campus football activities.

Third, our results discover that campus football injuries occurred, regardless of whether students participate in football activities alone or with the guidance of teachers. The highest number of injuries happened during football competition, followed by after-school football activities, while the lowest percentage occurred during football class instruction. Due to the intensity of the game and the fierce competition, students are subjected to a greater athletic load and the likelihood of injury increases, particularly when the competition's success or failure is at stake. Thus, it's unsurprising that football competition is the leading cause of campus football injuries, which is consistent with the existing literature demonstrating that injury occurrence is higher during competitions than during practice (28). Because after-school football activities are not supervised and restrained by teachers, students' safety awareness is low, making it simple to undertake risk-taking maneuvers and therefore increasing the likelihood of injury. In contrast, the football class has a united structure and administration of teachers, and the teacher's discipline and monitoring improves students' safety awareness, successfully lowering the injury occurrence. According to these findings, more qualified professionals, such as community-based football coaches capable of supervising after-school football activities, should be available to oversee training sessions and design exercise programs that match the needs, interests, and abilities of youth players.

Fourth, our results suggest a lack of safety awareness and injury prevention education is the primary cause of campus football injuries, and students and teachers alike should improve their safety knowledge and profession training. To our knowledge, injury prevention education in China is primarily delivered through lectures rather than practical training, and it is typically delivered infrequently, for example, once throughout an academic year via a classroom lecture. Proper education and adequate instruction are paramount in athletic injury prevention. As a result, we recommend that all campus football programs in China offer practical and need-based safety training for youth players and teachers in order to raise their safety knowledge. Relevant governing bodies of campus football programs could consider to adopt youth-tailed, proven effective injury prevention programs, such as FIFA “11+ Kids” (29) [note. FIFA 11 is not recommended (30)] and neuromuscular training (31). A nationally implemented injury prevention education program would not only benefit youth health while participating in campus football, but would also be monetarily beneficial to both youth and society (32, 33).

Fifth, our results reveal that that students' current level of first aid knowledge is concerning, with only 18.7% of students knowledgeable with basic first aid and only 11.4% familiar with CPR. The majority of students lack adequate first aid knowledge and skills, which corresponds to findings from other study of children and adolescents in China (34). This is not unrelated to the school's decision to offer first aid training to students and the quality of the training provided. According to a teacher survey, 62.5% of schools do not offer specialized first aid training, a trend that has also been observed in other regions of the world (35). It should be underlined that everyone, even children and adolescents, should be familiar with basic first aid and advanced first aid techniques such as CPR (36), which has been endorsed by the World Health Organization (37). As a result, schools should strengthen the development of first aid training courses, popularize first aid knowledge among students, and essentially require students and teachers to master pertinent first aid skills as a prerequisite to participate in campus football. Age-appropriate CPR teaching for children and adolescents should also be integrated into the school curriculum and relearned on a yearly basis. Recently, the Ministry of Education has decided to include first aid and CPR training in compulsory courses in middle and primary schools in China (38). A comprehensive emergency preparedness could enhance students', teachers', and schools' capability, resilience, and efficacy in the case of accidents and injuries during campus football activities.

Sixth, our results indicate that there is no clear solution in the event of campus football injuries. Campus football injuries are mostly the result of three distinct bodies of responsibility: students, teachers, and schools. Because sports injury can lead to emergency room visits and hospitalizations, lawful rights, interests, and liability arising from campus football injuries become a challenging legal issue (39, 40). While determination of the legal responsibility for athletic injuries is beyond the scope of the present study, we recommend seeking out third-party entities that would pay the school and its students' losses incurred as a result of athletic injuries. In principle, a commercial sports injury insurance covering school-related accidents could benefit students by easing financial burden imposed by the risk of campus football injuries and negotiating for follow-up care (41, 42).

There are a few limitations in this study. Because there is no national injury surveillance system for campus football, this study investigated epidemiological data using a survey approach. However, due to the retrospective nature of the injury data collection, the results may be skewed by recall bias. We would like to take this opportunity to urge that a national framework be established to monitor campus football injuries. Furthermore, because the study's participants were children and adolescents aged 9 to 18, and because kids in lower school grades had a poorer understanding of different types of injuries, we did not examine the injury severity. Both the occurrence and severity of injuries are critical for developing campus football programs, and hence we urge that future research employ systematic ways to analyze injury severity profiles and more detailed information about injuries from a clinical point of view.

Despite these limitations, we believe that our findings are applicable to youth participants from other regions of China because they were collected from a large student sample size participating in the Ministry of Education certified campus football programs in Ningxia. This is the first systematic investigation on the epidemiology of campus football injuries, and we envision that the findings would be carefully reviewed by schools, sports governing bodies (e.g., the Chinese Football Association), commercial entities, and the Ministry of Education in order to develop evidence-based injury prevention and management strategies that will promote the long-term development of Chinese youth football.

## Data Availability Statement

The raw data supporting the conclusions of this article will be made available by the authors, without undue reservation.

## Ethics Statement

The studies involving human participants were reviewed and approved by College of Education, Ningxia University, Yinchuan, China. Written informed consent to participate in this study was provided by the participants' legal guardian/next of kin.

## Author Contributions

HL, TB, and WJ: conceptualization, methodology, and project administration. HL, SH, TF, and LZ: formal analysis and writing—original draft preparation. TB and WJ: writing—review and editing and funding acquisition. All authors have read and agreed to the published version of the manuscript.

## Funding

This research was funded by Humanities and Social Science Research Project of the Ministry of Education: From the Game of Interests to the Research of Institutional Innovation: Review and Reflection on the History of Chinese Football Reform, grant number 19YJA890001 and the 2020 Hunan Provincial Social Science Fund Educational Special Project Key Project from Four in One to Sports Teaching Integration: A Study of Xi Jinping's Thoughts on School Physical Education in the New Era, grant number JJ209662.

## Conflict of Interest

The authors declare that the research was conducted in the absence of any commercial or financial relationships that could be construed as a potential conflict of interest.

## Publisher's Note

All claims expressed in this article are solely those of the authors and do not necessarily represent those of their affiliated organizations, or those of the publisher, the editors and the reviewers. Any product that may be evaluated in this article, or claim that may be made by its manufacturer, is not guaranteed or endorsed by the publisher.
